# Antisynthetase Syndrome Mimicking Pulmonary Infection: A Diagnostic Lesson From County‐Managed Hospital

**DOI:** 10.1002/ccr3.72085

**Published:** 2026-02-24

**Authors:** Jufen Cheng

**Affiliations:** ^1^ The Second Affiliated Hospital Zhejiang University School of Medicine The Second Affiliated Hospital Zhejiang University School of Medicine Kaihua Branch Kaihua County Zhejiang Province China

**Keywords:** antisynthetase syndrome, case report, old male, pulmonary infection

## Abstract

Antisynthetase syndrome (ASS) is a rare chronic autoimmune disease classified as a distinct subtype of idiopathic inflammatory myopathy, characterized by the presence of anti–aminoacyl‐transfer RNA synthetase (anti‐ARS) antibodies. Typical clinical features include myositis, interstitial lung disease (ILD), arthritis, fever, mechanic's hands, and Raynaud's phenomenon, with a predominance in middle‐aged to elderly females. We report an atypical case of a 75‐year‐old male presenting with a 6‐month history of anorexia and fatigue, followed by a 3‐week progression of chest tightness and dyspnea. Initially misdiagnosed as a pulmonary infection, the patient showed no improvement after two weeks of antimicrobial therapy. Diagnosis was ultimately confirmed through bronchoscopy and detection of anti–Jo‐1 antibodies. Subsequent corticosteroid treatment led to complete radiographic resolution and significant clinical improvement. This case underscores the importance of considering ASS in elderly male patients to prevent misdiagnosis and ensure timely intervention.

## Key Clinical Message

Antisynthetase syndrome can atypically present in elderly males with respiratory symptoms mimicking pulmonary infection. When antibiotic therapy fails, early serologic testing for anti‐synthetase antibodies (e.g., anti‐EJ) and bronchoscopy are essential. Prompt immunosuppressive treatment significantly improves outcomes in such cases.

## 1 Introduction

Antisynthetase syndrome (ASS) is a clinically heterogeneous autoimmune condition within the spectrum of idiopathic inflammatory myopathies (IIMs), pathologically characterized by the presence of highly specific anti‐aminoacyl‐tRNA synthetase autoantibodies [1]. These antibodies, which target enzymes essential for protein biosynthesis, are strongly associated with distinct clinical phenotypes [2]. Among them, anti‐Jo‐1 is the most prevalent, accounting for approximately 70%–80% of cases, whereas other subtypes—such as PL‐7, PL‐12, and EJ—exhibit lower frequencies and varying degrees of organ‐specific involvement [3]. The syndrome typically presents with a triad of interstitial lung disease (ILD), inflammatory myopathy, and polyarthritis, often accompanied by extrapulmonary manifestations including Raynaud's phenomenon, mechanic's hands, and unexplained fever [4, 5]. Although current diagnostic algorithms incorporate both serological markers and clinical features, the diagnosis of ASS remains challenging—particularly in primary care settings—due to its rarity, with an estimated incidence of 2–5 cases per 100,000 population [6].

The diagnostic complexity of ASS arises from several interrelated factors. First, respiratory symptoms—including dyspnea, nonproductive cough, and radiographic infiltrates—frequently mimic community‐acquired pneumonia, often leading to misdiagnosis and unnecessary antimicrobial therapy [7]. Second, the common dissociation between muscular and pulmonary involvement introduces diagnostic blind spots; approximately 20%–30% of patients present with isolated ILD in the absence of overt myositis [8]. Third, limited access to specialized immunologic testing in resource‐constrained settings delays antibody profiling, which remains essential for definitive diagnosis [9].

Delayed recognition of ASS increases the risk of irreversible pulmonary fibrosis, with studies indicating over 40% of associated interstitial lung disease cases progress within two years if untreated. This case, diagnosed through serology, imaging, and bronchoscopy after antibiotic‐refractory respiratory symptoms, demonstrates that timely immunosuppressive therapy can alter the disease course, highlighting the critical need to distinguish autoimmune from infectious processes for proper management.

This report serves a dual academic purpose: to enrich the phenotypic characterization of antibody‐negative ASS through comprehensive clinical documentation, and to propose a systematic diagnostic algorithm tailored for primary care physicians managing patients with unexplained respiratory and pulmonary syndromes. By integrating established diagnostic criteria with pragmatic adaptations for resource‐constrained settings, this study aims to shorten the median diagnostic delay—currently reported as 8 to 14 months in recent cohort studies—and thereby reduce the considerable morbidity associated with this heterogeneous autoimmune disorder.

## 2 Case History/Examination

A 75‐year‐old male was admitted to our department on October 15, 2022, presenting with a six‐month history of anorexia and generalized weakness, accompanied by chest tightness and dyspnea for the preceding three weeks. Approximately six months before admission, the patient developed a poor appetite and weakness of insidious onset, without associated fever, chills, chest discomfort, cough, sputum production, arthralgia, or rash. At that time, he did not seek medical evaluation. Three weeks before admission, he began experiencing progressive chest tightness and shortness of breath, without chest pain, hemoptysis, or syncope, prompting hospital presentation. Throughout the course, the patient remained conscious, exhibited a mildly decreased mood, maintained a generally normal diet and sleep pattern, and reported stable bowel and bladder function without significant weight change. His medical history was unremarkable for hepatitis, tuberculosis, diabetes mellitus, hypertension, cardiovascular or cerebrovascular diseases, surgeries, trauma, or known allergies. There was no notable personal, marital, or family history.

### 2.1 Physical Examination

The patient was alert but slightly lethargic. Vital signs were as follows: ear temperature 36.9°C, pulse rate 80 beats per minute, respiratory rate 19 breaths per minute, blood pressure 100/63 mmHg, oxygen saturation 96%, and blood glucose 6.9 mmol/L. Body weight was 72 kg. Respiratory examination revealed decreased breath sounds bilaterally with moist rales predominantly in the right lower lung field. Cardiovascular examination demonstrated a regular heart rhythm without pathological murmurs. The abdomen was soft and non‐tender, with no rebound tenderness. No edema was noted in the lower extremities. Musculoskeletal examination showed no swelling, tenderness, or deformities in the joints of all four limbs. No evident rash was observed on the limbs or trunk. Notably, Raynaud's phenomenon was present in both hands. Admission diagnosis: Pulmonary infection.

## 3 Investigations and Treatment

**FIGURE 1:**
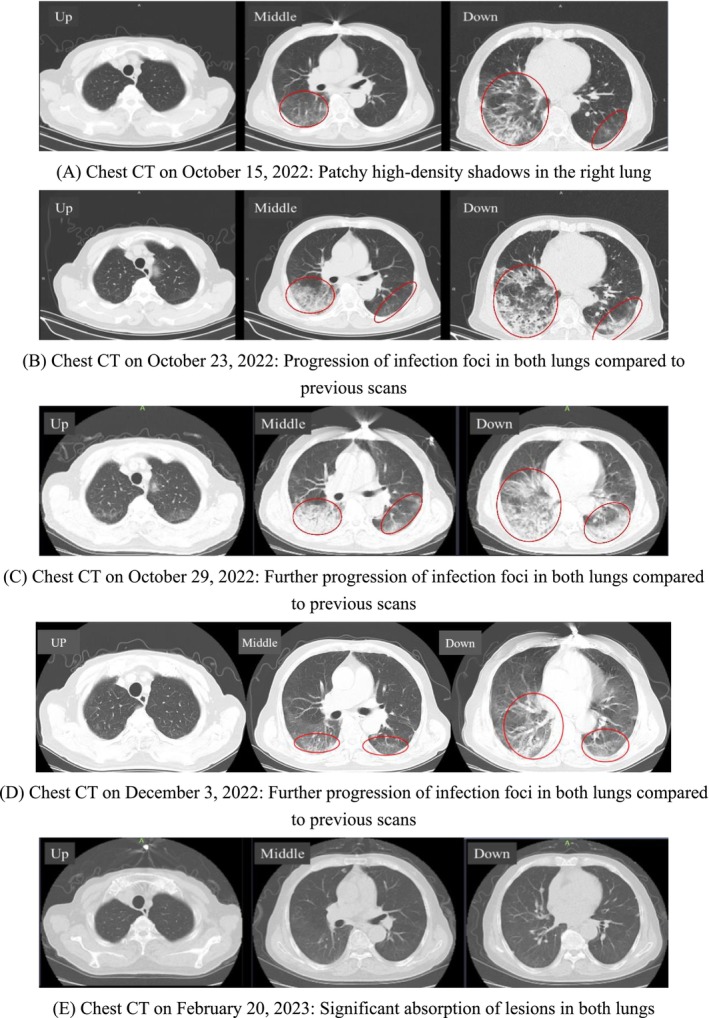
Evolution of the patient's chest CT. (A) Chest CT on October 15, 2022: Patchy high‐density shadows in the right lung. (B) Chest CT on October 23, 2022: Progression of infection foci in both lungs compared with previous scans. (C) Chest CT on October 29, 2022: Further progression of infection foci in both lungs compared with previous scans. (D) Chest CT on December 3, 2022: Further progression of infection foci in both lungs compared with previous scans. (E) Chest CT on February 20, 2023: Significant absorption of lesions in both lungs.

On admission on October 15, 2022, laboratory investigations revealed a white blood cell count of 8.30 × 10^9^/L with neutrophils constituting 64.5%, C‐reactive protein (CRP) at 9.19 mg/L, and procalcitonin at 0.06 ng/mL. Acid‐fast bacilli were not detected in sputum samples, and routine bacterial cultures identified normal oropharyngeal flora. The erythrocyte sedimentation rate (ESR) was elevated at 64 mm/h. Pathogen screening—including G‐test, GM‐test, *Mycoplasma pneumoniae* IgM, *Chlamydia pneumoniae* IgM, influenza A and B antigens, multiple respiratory virus antigens, SARS‐CoV‐2 nucleic acid, tuberculosis antibodies, and HIV serology—yielded negative results. Autoimmune serology showed a mildly elevated anti‐nuclear antibody (ANA) titer of 1:100. Chest computed tomography (CT) performed on October 15 (Figure 1A) showed patchy high‐density shadows in the lower lobe of the right lung.

**FIGURE 2:**
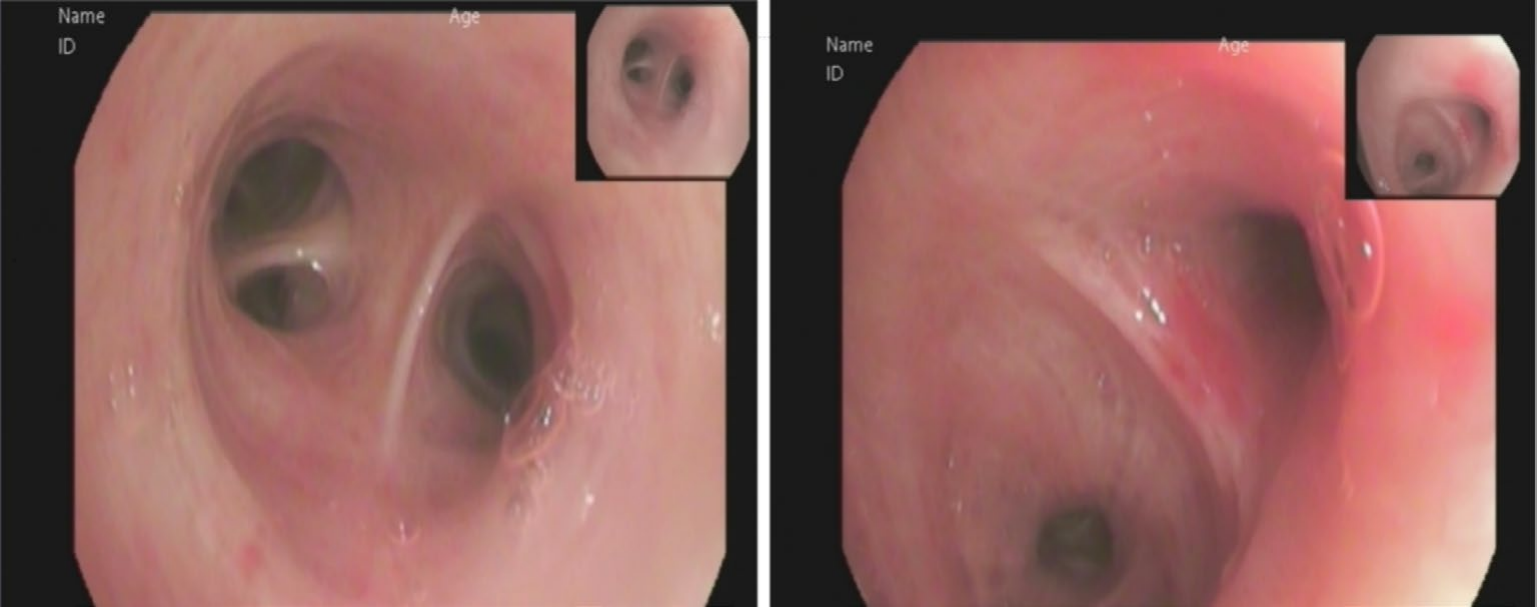
Physiological tracheal bifurcation under bronchoscopy. (Symmetric main bronchial orifices with smooth, pink mucosal lining)

Following admission, the patient received intravenous cefoperazone‐sulbactam sodium 2.0 g every 12 h for five days as empirical anti‐infective therapy. On October 20, laboratory tests revealed a white blood cell count of 8.74 × 10^9^/L, a neutrophil percentage of 70.8%, and an elevated high‐sensitivity C‐reactive protein (hs‐CRP) of 29.98 mg/L. Subsequently, cefoperazone‐sulbactam was discontinued, and oral moxifloxacin was initiated once daily for five days. However, chest computed tomography performed on October 23 (Figure 1B) demonstrated progression of pulmonary infiltrates compared with the scan obtained on admission (Figure 1A). On October 24, blood tests showed leukocytosis with white blood cells at 11.11 × 10^9^/L, neutrophils at 62.5%, red blood cells at 4.02 × 10^12^/L, and a further elevated hs‐CRP of 43.90 mg/L. Due to insufficient therapeutic response, moxifloxacin was discontinued on October 25 and replaced with intravenous piperacillin‐tazobactam 4.5 g every 8 h. Despite this escalation, laboratory findings on October 28 still indicated active inflammation: white blood cells 9.41 × 10^9^/L, neutrophils 75.0%, and CRP elevated to 69.61 mg/L. Clinically, the patient continued to report dizziness, chest tightness, and nocturnal productive cough with tenacious sputum, but no fever. Peripheral oxygen saturation measured on October 25 was 92%–93%, necessitating supplemental oxygen via nasal cannula. A repeat chest CT on October 29 (Figure 1C) revealed further deterioration of lung lesions. After two weeks of broad‐spectrum antibiotic therapy, the patient's clinical symptoms, laboratory markers, and radiological findings demonstrated continued progression, indicating failure of standard anti‐infective treatment. Bronchoscopic examination revealed no endobronchial masses (Figure 2). Bronchoalveolar lavage analysis, including cytology, microbiological testing, and metagenomic sequencing, yielded no evidence of malignancy, infection, or tuberculosis.

**FIGURE 3:**
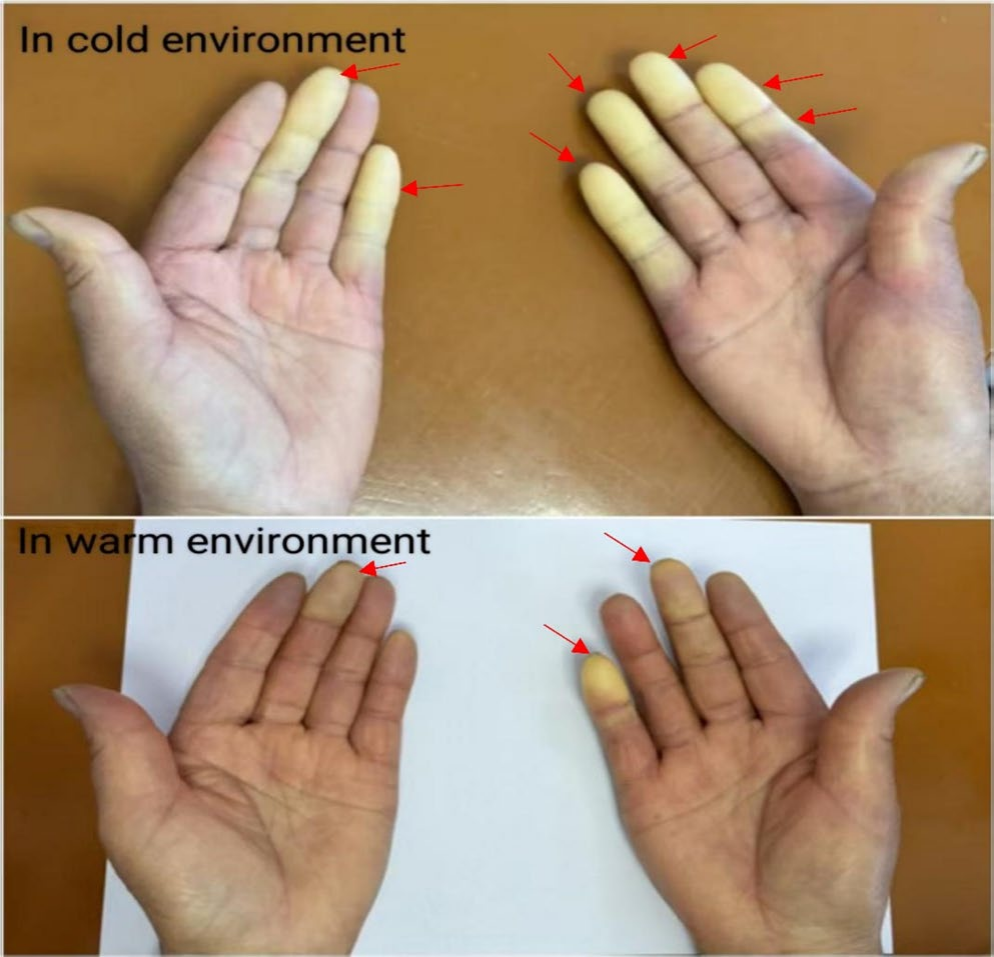
Bilateral Raynaud's phenomenon (in cold environment and warm environment).

The patient demonstrated no clinical improvement following prolonged anti‐infective therapy, prompting consideration of non‐infectious etiologies for the pulmonary lesions. Although elderly, the patient exhibited notably fewer facial wrinkles relative to age‐matched peers, alongside pronounced Raynaud's phenomenon affecting the fingers (Figure 3). Subsequent myositis‐specific antibody testing revealed positivity for anti‐EJ antibody, confirming a diagnosis of ASS. Initiation of corticosteroid therapy resulted in gradual amelioration of chest tightness and dyspnea. With supplemental oxygen, peripheral oxygen saturation improved to 95%. Serial laboratory evaluations demonstrated reductions in CRP and ESR. Correspondingly, chest imaging showed progressive resolution of pulmonary lesions (Figure 1D), with further substantial absorption confirmed on follow‐up imaging (Figure 1E).

### 3.1 Diagnosis

ASS (positive for anti‐EJ antibody); interstitial lung disease.

### 3.2 Treatment

The patient received intravenous methylprednisolone 40 mg twice daily for 10 days as anti‐inflammatory therapy, followed by a transition to oral prednisone at an initial dose of 15 mg once daily. After one week, the prednisone dose was tapered to 12 mg once daily, with gradual dose reduction thereafter to a maintenance level of 5 mg once daily. A tapering regimen of prednisone was followed, reducing the dose by 2.5 mg every 8 weeks. Lung function and CRP levels were measured monthly, and chest CT scans were performed every three months to monitor the disease course longitudinally and prevent relapse.

## 4 Conclusion and Results

### 4.1 Follow‐Up and Outcomes

After discharge, the patient continued oral prednisone therapy at 25 mg once daily. Regular outpatient follow‐ups were performed, and serial lung CT scans demonstrated significant resolution of pulmonary lesions, as detailed in Figure 1E.

## 5 Discussion

This case presented with fatigue, anorexia, chest tightness, dyspnea, and bilateral patchy opacities on chest imaging. Conventional anti‐infective therapies proved ineffective. Ultimately, through bronchoscopy to exclude infectious and neoplastic etiologies, combined with positive myositis‐specific antibody testing, the patient was diagnosed with ASS. Diagnostic criteria for ASS include the presence of serum anti‐synthetase antibodies along with at least one of the following clinical features: Raynaud's phenomenon, arthritis, interstitial lung disease (ILD), unexplained fever, and mechanic's hands (characterized by thickened and cracked skin on the fingertips) [10]. Existing data indicate a high prevalence of ILD among patients with ASS, which constitutes the primary contributor to morbidity and mortality in this population [11, 12]. To date, reports on ASS‐associated ILD have predominantly consisted of small‐sample retrospective studies and case reports.

Previous studies have demonstrated that among patients with ASS complicated by ILD, the median interval from symptom onset to initial diagnosis in rheumatology or pulmonology clinics is approximately 12.1 months (range: 0.5–120 months) [13]. In the present case, this interval was approximately seven months, underscoring the ongoing challenge of achieving early diagnosis. ASS predominantly affects middle‐aged and elderly females, with a male‐to‐female ratio of approximately 3:7, and the typical age of onset ranging from 40 to 50 years [14]. However, the patient described herein was a 75‐year‐old male, highlighting the potential for late‐onset disease in elderly men. The estimated annual incidence is 1 to 1.5 cases per million individual [15]. Moreover, geographic and ethnic variations have been observed; East Asian and African descent populations exhibit a relatively higher prevalence of non–anti‐Jo‐1 antibody positivity [16]. Clinical presentations such as cough and dyspnea are generally nonspecific, while the incidence of myositis‐associated muscle weakness is comparatively low. Given the nonspecific nature of respiratory symptoms, clinicians should maintain heightened vigilance for ASS in patients presenting with ILD as the initial manifestation, particularly when accompanied by cutaneous signs such as digital Raynaud's phenomenon [17]. Importantly, screening for anti‐synthetase antibodies should be considered even in the absence of overt myositis symptoms, including muscle weakness.

ILD is the most common extramuscular manifestation of ASS and constitutes a key clinical feature of ASS. It frequently occurs in adults with idiopathic inflammatory myopathies, particularly in patients with ASS and anti‐MDA5 antibody‐associated dermatomyositis [18]. The pulmonary involvement spectrum ranges from subclinical ILD to rapidly progressive respiratory failure. Concurrent myositis, characteristic cutaneous lesions, arthritis, and Raynaud's phenomenon are commonly observed. Testing for myositis‐specific and myositis‐associated antibodies facilitates diagnosis and disease stratification [5].

Common symptoms of ILD include cough, dyspnea, sputum production, respiratory difficulty, and cyanosis. Pleural effusion, when present, is usually minimal; significant effusions are rare. These clinical features are nonspecific [19]. ILD is also a frequent manifestation in anti‐EJ ASS. Fever affects over one‐third of patients during the disease course, with some studies indicating a higher incidence in patients positive for anti‐EJ or anti‐PL‐12 antibodies, although these findings require further validation [2]. Raynaud's phenomenon in ASS typically presents as digital vasospasm characterized by triphasic color changes—pallor, cyanosis, and erythema—triggered by cold exposure or emotional stress. It commonly coexists with ILD, myositis, arthritis, mechanic's hands (hyperkeratotic fingertips), and fever [2, 20]. Raynaud's phenomenon may be the initial manifestation or develop later during disease progression [21]. Its presence often reflects heightened disease activity or severity and can lead to serious vascular complications such as occlusive vascular disease and digital ischemia [22, 23]. In Jo‐1 antibody‐positive patients, Raynaud's phenomenon correlates with elevated creatine kinase levels and an increased risk of arthritis. Moreover, its association with ILD may influence prognosis [24, 25]. Given its clinical significance, Raynaud's phenomenon serves as an important marker in ASS, warranting close monitoring for vascular complications and disease progression.

In imaging evaluation, HRCT plays a pivotal role in characterizing disease extent and features in ASyS patients. Typical radiologic findings in ASyS‐ILD include ground‐glass opacities (GGOs), alveolar consolidations, traction bronchiectasis, reticular patterns, thickened interlobular septa, and volume loss, reflecting underlying interstitial involvement. These abnormalities predominantly affect the lower lobes, with distribution often peripheral or along bronchovascular bundles, and may occasionally be diffuse. Patients with anti‐EJ‐associated ILD frequently exhibit hazy opacities localized mainly in the lower lobes, with nonspecific interstitial pneumonia (NSIP) commonly representing the initial ILD pattern. Chest imaging and histopathological analyses most frequently reveal NSIP and organizing pneumonia patterns [26, 27].

The initial characterization of anti‐synthetase antibodies centered on their association with myositis, ILD, and mixed connective tissue disease (MCTD), giving rise to the concept of the ASyS triad. Nevertheless, clinical presentations of ASyS are heterogeneous. Patients lacking the full triad may present with isolated organ involvement or varied combinations of symptoms as their initial manifestation. Therefore, early serum autoantibody testing, comprehensive pulmonary imaging, and careful assessment of extrapulmonary features are critical for timely diagnosis and management [28, 29].

### 5.1 Treatment of ASS

Currently, no universally effective therapeutic regimen exists that simultaneously addresses ILD, myositis, and polyarthritis. Corticosteroids remain the first‐line treatment; however, prolonged use necessitates adjunctive immunosuppressants to mitigate adverse effects. Refractory cases often require combination therapy, and patients unresponsive to conventional regimens may benefit from steroid pulse therapy in conjunction with rituximab or intravenous immunoglobulin (IVIG). Early identification is essential—patients presenting with unexplained fever, muscle weakness, or respiratory distress should undergo prompt testing for anti‐synthetase antibodies to facilitate timely intervention [28, 30]. Variations in antibody profiles, such as anti‐PL‐12 and anti‐Ro52, may influence clinical phenotypes and therapeutic responses [31]. Management of ASS demands a multidisciplinary approach centered on immunosuppression, complemented by targeted therapies and supportive care [32, 33]. Future studies are warranted to develop standardized treatment protocols, particularly addressing refractory ILD and antibody‐specific strategies. Furthermore, for patients with refractory disease or those who experience relapse during steroid tapering, the introduction of other immunosuppressive agents should be considered. IL‐6 inhibitors (e.g., tocilizumab) represent a promising therapeutic option. Emerging evidence from case reports and series indicates that tocilizumab can be effective in controlling disease activity, particularly in cases with progressive interstitial lung disease, when conventional therapies fail [34, 35]. Therefore, had our patient shown an inadequate response to corticosteroids or relapsed, the initiation of an IL‐6 inhibitor would have been a rational next‐step strategy.

The uniqueness of this case is reflected in several aspects:

1. The patient's demographic profile is atypical, as ASS‐ILD predominantly affects middle‐aged women. This underscores the necessity for clinicians to maintain vigilance for atypical presentations, recognizing that ASS‐ILD can also manifest in elderly males, often with nonspecific symptoms mimicking infectious pulmonary diseases.
2. The identification of anti‐EJ antibodies highlights a distinct clinical phenotype and prognostic implication. Anti‐EJ antibodies are relatively rare within the anti‐synthetase antibody spectrum, and their association with specific clinical features and outcomes remains incompletely understood. This case suggests that anti‐EJ–positive patients may predominantly present with ILD and exhibit rapid disease progression, warranting early recognition and aggressive therapeutic intervention.
3. Timely diagnosis and prompt initiation of appropriate treatment are critical for optimizing outcomes in ASS‐ILD. The initial misdiagnosis delayed immunosuppressive therapy, resulting in ineffective antibiotic use. Therefore, in patients suspected of ILD—particularly those refractory to antibiotics—comprehensive history, physical examination, and early autoantibody screening are imperative to establish diagnosis and facilitate early immunosuppressive management.

This case underscores that ASS‐ILD may occur in elderly males with atypical presentations, rendering it susceptible to misdiagnosis. Enhanced clinical awareness, early autoantibody screening, and bronchoscopy are essential to enable timely diagnosis and treatment, thereby improving patient prognosis.

## Author Contributions

**Jufen Cheng:** conceptualization, data curation, formal analysis, investigation, investigation, methodology, methodology, project administration, project administration, resources, resources, software, software, supervision, supervision, validation, validation, visualization, visualization, writing – original draft, writing – original draft, writing – review and editing, writing – review and editing.

## Funding

The author has nothing to report.

## Ethics Statement

Ethics committee approval was not required for this case report in accordance with the institutional policies, as it presents a single anonymized patient case with informed consent.

## Consent

Patient consent to publish clinical information and images was obtained in written form using a consent form in Chinese. The patient has agreed to the terms outlined in Wiley's standard consent form, including understanding that their medical information will be published on an open access basis and may be freely accessed worldwide.

## Conflicts of Interest

The author declares no conflicts of interest.

## Data Availability

The data that support the findings of this study are available from the corresponding author upon reasonable request.
